# Fair and Dark

**Published:** 1921-02-12

**Authors:** 


### FAIR AND DARK.

The colour index of these islands is a character of much anthropological and general interest, which from time to time has had much attention bestowed upon it. In the *Journal of the Royal Anthropological Institute* there has recently been published a recension of results obtained in this field of work, by Professor F. G Parsons. This anthropologist agrees in the main with the classic findings of Beddoe, and it is gratifying to reflect that both these scientists are, like several other anthropologists, such as Drs. Seligmann and W. H. R. Rivers, of the medical profession. However, a careful review of Beddoe's tables, aided by some fresh work, suggests a few corrections and new conclusions. An important fallacy to be borne in mind as a preliminary is that almost all the agencies acting on the hair of the lower orders tend to darken it, *e.g.* grease, sweat, and dirt; so that an observer will usually do well to lean towards the lighter rather than the darker tint in difficult cases. What soon strikes every worker at colour index is that children are unsatisfactory subjects, because the hair of children tends to darken much with age.

### Infantile Changes in Colour.

Some fair-haired children are potential brunettes, as shown already by the tint of their eyes and complexion. The eyes themselves do not change much after the first year. Within that period, however, they change a good deal. Many young parents are quite aware of the second fact, but not of the first one. The truth seems to be that almost all English infants have at birth eyes of a hue the author happily describes as lead-blue; less than one per cent. are born with dark eyes; indeed the sister of the maternity ward at St. Thomas's Hospital is quoted as having seen only two such cases. The lead-coloured eye has a faint ring of white surrounding the pupillary margin, and if the eye is going to be dark, then the change becomes evident by the third month.

A new finding of considerable interest is that women all over this country are appreciably darker than men. It is surmised that as the constancy of this difference is so great there must be a definite reason for it. However, the only etiological factor suggested is not a very convincing one—namely, that "women are more evenly distributed over the country than men." The sex-excess of darkness is greater in the north than elsewhere. Obviously this result vitiates calculations as to the nigrescence of any particular region which do not take the question of sex-incidence into account.

### Complexion and Locality.

The West of England is notable for dark hair, and also the West of Ireland. These investigations, as the author points out, certainly negative Professor Keith's assertion that representative groups from, say, Sligo and Norfolk, would show no important physical differences. Agreeably with Ammon's Law (though not so stated), urban dwellers are found to be of darker coloration than rural ones. Red hair is described as a little commoner in the upper social classes than in the lower ones, at least as regards London and Bristol. Generally, dark characters are attributed to preponderance of Mediterranean-derived stocks, and fair to Nordic blood.

The paper is interesting, although the author makes no reference to Hurst's Mendelian criterion of eye-pigmentation, and takes but small account of the work done on red hair—much of which is to be found set out in the admirable monograph of Pearson, Nettleship, and Usher on albinism in man. It is, however, professedly tentative in one or two directions. Work of this kind is especially valuable because the opportunity for it is passing. Improved means of locomotion (even in the last ten years the motor-bus and motor-cycle, for instance) lead to migration of stocks and obliteration of autochthonous distinctions. In some localities the common local names will often be the same as those in Domesday Book, but in the majority they will be quite different; and this majority tends to increase. The riddle of race has always been a hard one in these islands, and it is to be feared it grows harder as time goes on. The more reason for more inquiry.

